# Folate levels in hepatocellular carcinoma patients with portal vein thrombosis

**DOI:** 10.1186/s12876-020-01525-3

**Published:** 2020-11-10

**Authors:** Giulia Malaguarnera, Vito Emanuele Catania, Saverio Latteri, Antonio Maria Borzì, Gaetano Bertino, Roberto Madeddu, Filippo Drago, Michele Malaguarnera

**Affiliations:** 1 Department of Biomedical and Biotechnological Science, University of Catania, 95123 Catania, Italy; 2 Department of Medical, Surgical Sciences and Advanced Technologies “G.F. Ingrassia”, University of Catania, Via Santa Sofia 78, 95123 Catania, Italy; 3 Research Centre “The Great Senescence”, University of Catania, 95120 Catania, Italy; 4 Hepatology Unit, Department of Clinical and Experimental Medicine, University of Catania, Policlinico “G. Rodolico”, Catania, Italy; 5 Department of Biomedical Sciences, University of Sassari, Sassari, Italy

**Keywords:** Folate, Hepatocellular carcinoma, Thrombosis, Portal vein thrombosis, Homocysteine, Red cell folate

## Abstract

**Background:**

Portal vein thrombosis (PVT) occurs frequently in hepatocellular carcinoma (HCC) and is often diagnosed in the course of a routine patient evaluation and surveillance for liver cancer. The purpose of this study is to investigate the relationship between folate status and portal vein thrombosis.

**Methods:**

HCC with PVT patients were 78, HCC without PVT were 60 and control subjects were 70 randomly selected. We evaluate serum and red blood cellular folate, homocysteine, alpha fetal protein cholesterol, triglycerides, prothrombin time.

**Results:**

HCC patients with PVT showed lower levels of serum folate, respect HCC patients without PVT, with an average difference of 1.6 nmol/l *p* < 0.01 (95% CI − 2.54 to − 0.66), red cell folate 33.6 nmol/l *p* < 0.001 (95% CI − 43.64 to − 23.55) and albumin 0.29 g/dl *p* < 0.001 (95% CI − 0.42 to − 0.15); PVT patients displayed higher levels of bilirubin 0.53 mg/dl *p* < 0.001 (95% CI 0.23 to 0.78), INR 0.91 *p* < 0.001 (95% CI 0.72 to 1.09), γGT 7.9 IU/l (95% CI 4.14 to 11.65) and homocysteine 4.6 μmol/l *p* < 0.05 (95% CI 0.32 to 8.87)

**Conclusion:**

The low folate concentration and higher levels of homocysteine are associated with the loss of antithrombotic function, and with a more aggressive course of HCC and with a higher change of complications related to portal vein thrombosis

## Background

Hepatocellular carcinoma (HCC) is one of the leading causes of cancer mortality [1].

There are striking variations in its incidence in various parts of the world, although the 83% of the total deaths occur in developing countries [2, 3].

Many studies have focused on the investigation of markers to predict the outcomes of HCC and to exploring accurate prognostic factors for the clinical examination [4, 5]. Many HCC patients display portal hypertension, which cause the blood flow velocity of the portal vein to slow down. Changes in haemodynamic of the portal vein are the anatomical base of Portal Vein Thrombosis (PVT) formation. This complication is a multifactorial process, characterized also by the increase of systemic prothrombotic factors and the presence of local inflammatory foci. As a result, there is hypercoagulation, accompanied by the slowing of blood flow and damage to the vessel walls [6–8].

Patients with HCC with PVT have poor prognoses than those without PVT [9–11].

Evidence show a relationship between nutrition and environmental factors in relation to the progression of HCC. For instance, folate (known also as vitamin B9) and its derivatives are found abundantly in leafy green vegetable and fresh fruit. These compounds play an essential role in the production and maintenance of new cells and is involved in DNA methylation, DNA synthesis and DNA repair.

Folate is a water-soluble beta-vitamin critical for health. It participates in numerous single-carbon exchange reactions that are essential for the synthesis of nucleotides purine/pyrimidine. Folates have been studied as a potential chemo-preventive agent.

Dietary folate is digested in the jejunal brush border surface by intestinal glutamate carboxy peptidase, followed by the transfer, through the portal vein to the liver where it is transported across membranes by the reduced folate carrier, possibly together with folate binding protein or the proton coupled folate transporter. These molecules are mainly stored in the liver, where are used in several reaction. Then, dietary folate is transported to the systemic blood circulation and in part, eliminated by the biliary excretion [12].

Both dietary and endogenous folate plays an important role in epigenetic methylation reactions, in lipid export, antioxidant defence and the hepatic methionine metabolism, which in turn regulates homocysteine levels [13, 14].

The objective of our study is to investigate, the relationship among blood folate and PVT in HCC patients, comparing these variables in controls subjects and in patients with and without PVT.

## Methods

### Patients recruitment

The subject with HCC included in this study were 138 patients with HCC: 78 HCC with PVT (31 females and 47 males) aged 65 or more years, and 60 HCC without PVT (24 female and 36 males), aged 60 or more years, living in Sicily (Italy).

The control subjects were 70 (35 female/35 males), aged 60 years or more. They were randomly selected and healthy without any history of cancer, major organ failure or active intravenous drug abuse.

The inclusion criteria were patients older than 18, with histologically proven HCC and with written informed consent.

This study used similar recruitment strategies, inclusion and exclusion criteria and measurement protocol adopted in previous studies on HCC patients with PVT and in controls subjects.

The questionnaires were administered by trained interview and focused on dietary, sociodemographic, environmental and lifestyle information.

### Serum and whole blood folate assay

Serum and whole blood samples were collected into citrate tubes during the screening visit. They were immediately put on ice and centrifuged (2000×*g*, 4 °C, 10 min) within 60 min. Both serum and whole blood samples were stored at − 70 °C until analysis and were measured using QuantaPhase radioassay II Kit (Bio-Rad Laboratories, Hercules, California).

### Statistical analysis

Data were expressed as mean ± standard deviation. To assess relationship and differences between folate, Hcy, alfa-fetoprotein of different patent groups, parametric, non-parametric Fisher's exact test and 95% CI Odds Ratio (O.R.) Baptista–Pike method or ordinary one-way ANOVA with Tukey's multiple comparisons test. A *p* value < 0.05 was considered statistically significant. Data were analysed using the GraphPad Prism 8 statistical software package (8.4.2 Macintosh Version; GraphPad Software San Diego, CA).

Univariate and multivariate linear regression were built to assess the relationship between folate concentration and sociodemographic data, area of residence (urban, metropolitan, semi urban, rural), social class (managers, skilled, semiskilled, unskilled) and clinical features of HCC patients.

## Results

PVT patients’ ages ranged between 60 years and over; of these 35.90% were smokers, 12.82% were diabetics, 12.82% complained of renal failure; 15.38% of Hypertension; 23.08% of cardiovascular disease; 19.23% of dyspepsia, 12.82% experienced alcohol consumption. Etiologic factors in PVT patients were 44.87% for HCV, 32.5% for HBV and 10.26% unknown (Tables 1 and 2).

**Table 1:** Demographic and baseline characteristics of the study population

	With PVT	NPVT	Controls	PVT versus NPVT		PVT versus Control		NPVT versus Control	
	78 pt	60 pt	70 pt	*p *value	95% CI	*p *value	95% CI	*p *value	95% CI
Age (range)	60–75	60–75	60–75	–	–	–	–	–	–
Female/male	31/47	24/36	35/35	> 0.9999	0.5042 to 1.968	0.2475	0.3352 to 1.275	0.2913	0.3264 to 1.326
BMI (Kg/m^2^)	23.8 ± 4.4	25.0 ± 4.2	24.7 ± 4.2	0.2336	− 2.934 to 0.5336	0.409	− 2.562 to 0.7621	0.9161	− 1.476 to 2.076
Systolic blood pressure (mmHg)	140.2 ± 11.4	137.0 ± 12.8	140.1 ± 14.9	0.3293	− 2.097 to 8.497	0.9988	− 4.978 to 5.178	0.3700	− 8.527 to 2.327
Diastolic blood pressure (mmHg)	82.2 ± 8.4	81.8 ± 9.2	81.4 ± 9.7	0.9644	− 3.283 to 4.083	0.8543	− 2.731 to 4.331	0.9661	− 3.373 to 4.173
Heart rate (b.p.m)	83.7 ± 9.68	83.8 ± 9.7	81.4 ± 10.2	0.9981	− 4.099 to 3.899	0.3344	− 1.534 to 6.134	0.3517	− 1.697 to 6.497

CI, confidence interval; pt, patients; bpm, beats per minute; BMI, Body Mass Index

**Table 2:** Characteristics of the patients and risk factors

	With PVT = 78 pt		NPVT = 60 pt		Controls = 70 pt		PVT versus NPVT			PVT versus control			NPVT versus control		
	N	%	N	%	N	%	*p*	95% CI O.R.	Sig	*p*	95% CI O.R.	Sig	*p*	95% CI O.R.	Sign
Smokers/no smokers	28	35.90	27	45.00	20	28.57	0.2975	0.3451 to 1.347	ns	0.3821	0.7087 to 2.877	ns	0.0672	0.9742 to 4.156	ns
Diabetes mell	10	12.82	9	15.00	8	11.43	0.8049	0.3208 to 2.078	ns	> 0.9999	0.4387 to 3.093	ns	0.6078	0.5114 to 3.884	ns
Hypertension	12	15.38	14	23.33	10	14.29	0.2757	0.2682 to 1.431	ns	> 0.9999	0.4575 to 2.783	ns	0.2569	0.7175 to 4.602	ns
Renal failure	10	12.82	12	20.00	12	17.14	0.3484	0.2274 to 1.425	ns	0.4952	0.2786 to 1.697	ns	0.8211	0.5195 to 2.809	ns
Cardiovascular dis	18	23.08	16	26.67	10	14.29	0.6921	0.3726 to 1.791	ns	0.2096	0.8020 to 4.219	ns	0.1226	0.9064 to 5.360	ns
Dyspepsia	15	19.23	21	35.00	12	17.14	0.0501	0.2122 to 0.9495	ns	0.8325	0.5049 to 2.610	ns	0.026	1.132 to 5.798	*
Alcohol	10	12.82	16	26.67	0	0	0.0489	0.1659 to 0.9651	*	0.0016	–	**	< 0.0001	–	****
HCV	35	44.87	25	41.67	0	0	0.732	0.5902 to 2.235	ns	< 0.0001	–	****	< 0.0001	–	****
HBV	25	32.05	9	15.00	0	0	0.028	1.135 to 6.349	*	< 0.0001	–	****	0.0007	–	***
HCC unknown aetiology	8	10.26	10	16.67	0	0	0.3132	0.2084 to 1.480	ns	0.007	–	**	0.0003	–	***

Fisher's exact test, 95% CI Odds Ratio (O.R.) Baptista–Pike method

pt, patients; HCC, hepatocellular carcinoma; HCV, hepatitis C virus; HBV, hepatitis B virus

Summary: *< 0.05; **< 0.005; ***< 0.001; ****< 0.0001

HCC without PVT patient’s ages ranged 60 years and over; of these 45% were smokers or previous smokers, 15% were diabetics, 23.33% complained of hypertension, 26.67% of cardiovascular disease, 20% of renal failure, 35% of dyspepsia, 26.67% of alcohol consumption. Etiologic factors were 41.67% HCV, 15% HBV, 16,67% unknown.

In the control group ages ranged between 60 years and over; of these 28.57% were smokers, 11.43% were diabetics, 14.29% complained of hypertension, 14.29% of cardiovascular disease, 17.14% of renal failure, 17.14% of dyspepsia (Tables 1 and 2).

### Comparison from HCC patients with PVT to HCC patients without PVT

We observed that were lower serum folate 1.6 nmol/l *p* < 0.01 (95% CI − 2.54 to − 0.66), red cell folate 33.6 nmol/l *p* < 0.001 (95% CI − 43.64 to − 23.55), albumin 0.29 g/dl *p* < 0.001 (95% CI − 0.42 to − 0.15); were higher bilirubin 0.53 mg/dl *p* < 0.001 (95% CI 0.23 to 0.78), INR 0.91 *p* < 0.001 (95% CI 0.72 to 1.09), γGT 7.9 IU/l (95% CI 4.14 to 11.65) and homocysteine 4,6 μmol/l *p* < 0.05 (95% 0.32 to 8.87) (Table 3).

**Table 3:** Laboratory parameters and classifications of subjects included in the study

	PVT (n = 78)	NPVT (n = 60)	Controls (n = 70)	PVT versus NPVT(*p* value)	PVT versus Controls (*p *value)	NPVT versus Controls (*p *value)
Bilirubin (mg/dl)	2.87 ± 0.6	2.36 ± 1.02	1.24 ± 0.38	< 0.001	< 0.001	< 0.001
Albumin (g/dl)	3.15 ± 0.42	3.44 ± 0.38	3.96 ± 0.55	< 0.001	< 0.001	< 0.001
INR	2.91 ± 0.54	2.00 ± 0.57	1.22 ± 0.36	< 0.001	< 0.001	< 0.001
Tot cholesterol (mmol/l)	5.77 ± 1.08	5.69 ± 1.01	5.50 ± 1.04	0.659	0.124	0.297
HDL (mmol/l)	1.40 ± 0.36	1.41 ± 0.37	1.46 ± 0.40	0.873	0.338	0.463
LDL (mmol/l)	3.44 ± 0.38	3.48 ± 0.39	3.46 ± 0.37	0.545	0.747	0.765
Triglycerides (mmol/l)	1.57 ± 0.70	1.50 ± 0.68	1.48 ± 0.65	0.556	0.421	0.864
ALT (IU/l)	51.20 ± 10.80	48.20 ± 11.90	34.50 ± 10.20	0.124	< 0.001	< 0.001
AST (IU/l)	49.20 ± 10.60	48.70 ± 10.80	33.10 ± 10.80	0.786	< 0.001	< 0.001
γGT (IU/l)	82.80 ± 10.20	74.90 ± 12.10	27.40 ± 10.80	< 0.001	< 0.001	< 0.001
AFP (mg/l)	236.10 ± 15.80	240.20 ± 14.20	3.20 ± 0.60	0.117	< 0.001	< 0.001
Serum folate (nmol/l)	5.24 ± 2.81	6.84 ± 2.71	8.50 ± 3.01	< 0.001	< 0.001	0.001
Red cell folate (nmol/l)	144.20 ± 30.60	177.80 ± 28.20	294.20 ± 27.80	< 0.001	< 0.001	< 0.001
Total homocysteine (μmol/l)	32.80 ± 12.20	28.20 ± 13.10	10.70 ± 6.90	0.035	< 0.001	< 0.001

PVT, portal vein thrombosis; INR, International normalized ratio; HDL, high-density lipoprotein; LDL, low-density lipoprotein; ALT, alanine transferase; AST, aspartate transferase, γGT, gamma-glutamil-tranferase

### Comparison between HCC patients with PVT and controls

We observed that were lower serum folate 3.28 nmol/l *p* < 0.001 (95% CI − 4.20 to − 2.31), red cell folate 150 nmol/l *p* < 0.001 (95% CI − 159.53 to − 140.46), albumin 0.83 g/dl *p* < 0.001 (95% CI − 0.96 to − 0.65); were higher bilirubin 1.63 mg/dl *p* < 0.001(95% CI 1.46 to 1.79), INR 1.69 *p* < 0.001 (95% CI 1.54 to 1.84) ALT 16.70 IU/l *p* < 0.001 (95% CI 13.27 to 20.11) AST 16 IU/l *p* < 0.001 (95% CI 12.62 to 19.58) γGT 55.4 IU/l *p* < 0.001 (95% CI 51.28 to 58.81), αFP 232.9 mg/l *p* < 0.001 (95% CI 229.16 to 236.63) and homocysteine 22.10 μmol/l *p* < 0.001 (95% CI 51.28 to 58.81). The results are showed in Table 3

### Comparison between HCC patients without PVT and controls (Table 3)

We observed that serum folate was lower 1.66 nmol/l *p* < 0.001 (95% CI − 2.66 to − 0.66), red cell folate 116.4 nmol/l *p* < 0.001 (95% CI − 126.14 to − 106.66), Albumin 0.52 g/dl *p* < 0.001 (95% CI − 0.68 to − 0.35); Bilirubin was higher 1.12 mg/dl *p* < 0.001(95% CI 0.86 to 1.38), INR 0.78 *p* < 0.001 (95% CI 0.61 to 0.94) ALT 13.70 IU/l *p* < 0.001 (95% CI 9.86 to 17.53) AST 15.60 IU/l *p* < 0.001 (95% CI 11.84 to 19.36) γGT 47.50 IU/l *p* < 0.001 (95% CI 43.52 to 51.47), αFP 237.00 mg/l *p* < 0.001 (95% CI 233.64 to 240.36), total homocysteine 17.50 μmol/l *p* < 0.001 (95% CI 13.93 to 21.06).

The median value of serum folate levels was 5.9 nmol/l. Patient were divided into two groups each of 69 patients (Table 4): ≤ 5.9 nmol/l and > 5.9 nmol/l.

**Table 4:** Clinical features of the HCC patients

	Serum folate				*p* value	Signif	Odds-ratio	95% CI O.R.
	≤ 5.9 mm/l (69 pt)		> 5.9 mm/l (69 pt)					
	N	%	N	%				
PVT	44	63.77	34	49.28	0.1219	ns	1.812	0.9335 to 3.584
HCC stage I	16	23.19	20	28.99	0.5612	ns	0.7396	0.3337 to 1.573
HCC stage II	18	26.09	23	33.33	0.4564	ns	0.7059	0.3412 to 1.519
HCC stage III	20	28.99	21	30.43	> 0.9999	ns	0.9329	0.4610 to 1.876
HCC stage IV	15	21.74	5	7.25	0.0276	*	3.556	1.265 to 9.289
Clip Score 0–2	31	44.93	28	40.58	0.7309	ns	1.195	0.6229 to 2.310
Clip Score 3–6	38	55.07	41	59.42	0.7309	ns	0.8371	0.4329 to 1.605
Cirrhosis	25	36.23	33	47.83	0.2272	ns	0.6198	0.3139 to 1.194
< 50 mm	25	36.23	33	47.83	0.2272	ns	0.6198	0.3139 to 1.194
> 50 mm	44	63.77	36	52.17	0.2272	ns	1.613	0.8373 to 3.185
Tumour Invasion	26	37.68	32	46.38	0.3886	ns	0.6991	0.3570 to 1.345
Vascular Invasion	29	42.03	31	44.93	0.8637	ns	0.8887	0.4619 to 1.701
Extra-hepatic MTS	44	63.77	29	42.03	0.0166	*	2.428	1.190 to 4.875

PVT, portal vein thrombosis; HCC, hepatocellular carcinoma; Mtd, max tumour diameter

Summary:*< 0.05; **< 0.005; ***< 0.001; ****< 0.0001

We observed deficient serum folate levels in PVT patients in high grade (IV stage) of HCC (*p* = 0.028) and in presence of extrahepatic metastases (*p* = 0.016) (Table 4).

To identify the prognostic factors of PVT both univariate and multivariate analyses were used to evaluate red cellular folate and other clinical pathological variables.

The results suggested that red cellular folate (*p* < 0.01), higher homocysteine level (*p* < 0.01) and higher AFP (*p* < 0.05) were related to PVT. The other variables included in multivariate models were not statistically significant.

## Discussion

Serum folate, RBC folate and plasma total Hcy are the three most used biochemical indicators to assess folate status [15].

In our study we observed that serum folate and red cells folate in HCC patients with PVT were lower than in no PVT and controls subjects, whereas homocysteine was higher than in no PVT and in control subjects. These conditions induce atherosclerotic and thrombotic vascular disorders and promote oxidative stress, inflammation and endothelial injury, as well prothrombotic effect. Portal vein thrombosis is a relatively frequent event in cirrhosis and HCC, where its incidence varies from 7.4 to 17.8% in different studies [16–18].

In PVT, the concentration of folate in red cells result as a strong indicator of folate status, because the transitory changes of the diet do not control it. The concentration of folate in red cells is much higher than in plasma. Moreover, its concentration is established during erythropoiesis, for this reason its levels last for approximately 4 months [19].

The patients with PVT and HCC display an aggressive disease course, worsening of liver conditions, complications related to portal hypertension, low tolerance to treatment and lower serum folate and HHCy if compared with HCC subject without PVT [20–23].Numerous variables such as agents, drugs, diseases and lifestyle factors have a relevant impact on folate status. Especially those substance that act directly or indirectly on enzymes where folates operate as cofactors, but also as a consequence of the increase of disulphide exchange reactions, or the impairment of folate absorption. In fact, folate deficiency may be caused by the inadequate intake, the reduced absorption from the gastrointestinal tract, or by increased drug consumption and interactions. Subjects without a balanced diet, patients with renal disease, inflammatory bowel disease or malignant disease are more at risk for folate deficiency and, as a consequence, they have elevated levels homocysteine [24–29].

The role of folate deficiency and the hyperhomocysteinemia in PVT and in venous thromboembolism deserve also a relevant attention to evaluate the role of these findings in the pre-operative, operative and post-operative thrombotic complication of HCC.

PVT prophylaxis with anticoagulants is still controversial and relatively less investigated; for this reason, it can be recommended on an individual basis. Despite these results, the reduction of thromboembolism with folate supplementation in patients with PVT and HCC is scarce [30–32].

The identification of the high-risk patients with hypofolatemia and/or hyper homocysteinemia, through the evaluation of genetic mutation and nutritional deficiency is essential to plan clinical management in subjects who are candidate for major surgery or liver transplantation [33].

Folate carry out their metabolic functions when it is converted to 5-methyl tetrahydrofolate-. 5-methyltetrahydrofolate and it is associated with improvements in NOS coupling and nitroxide (NO) synthesis in vivo [34–38].

NO is a potent vasodilator that improves vascular function through its anti-inflammatory, antithrombotic and anti-angiogenic properties.

The study has established an inverse association between the folate level and tumour size, multiplicity and metastases; disease progression was categorized into stages 1 to 4, and serum folate decreased as disease stage progressed [39–43] (Table 4).

Therefore, folate deficiency aids the incorporation of uracil into DNA, which can lead to DNA breaks and chromosome instability; such breaks could contribute to the increased risk of cancer [44–49].

## Conclusion

Our study supports the hypothesis that the folate status shows a protective role in HCC prevention, development and prognosis.

Further longitudinally designed and interventional studies with a large sample size may help to determine the optimal strategies to improve folate status of HCC patients [50] and to evaluate the effects in coagulation disorders in HCC patients with PVT [51].

## Data Availability

The datasets used and/or analysed during the current study available from the corresponding author on a reasonable request.

## Abbreviations

PVT: Portal vein thrombosis
HCC: Hepatocellular carcinoma
γGT: γ-Glutamyltransferase
DNA: Deoxyribonucleic acid
αFP: Alpha-fetoprotein
CT: Computed tomography
MR: Magnetic resonance
CLIP: Cancer Liver Italian Program score
TNM: (Tumour Nodes Metastases) Classification of Malignant Tumours
US: Ultrasonography
°C: Degree Celsius
G: Gravitational acceleration
RBC: Red blood cellular folate
tHcy: Total homocysteine
HHcy: Hyperhomocysteine
HCV: Hepatitis C virus
HCB: Hepatitis B virus
INR: International normalized ratio
AST: Aspartate transaminase
ALT: Alanine transaminase
BMI: Body Mass Index
HDL: High-density lipoprotein
LDL: Low-density lipoprotein
NO: Nitroxide
